# Effect of silver nanoparticles on the standard soil arthropod *Folsomia candida* (*Collembola*) and the eukaryote model organism *Saccharomyces cerevisiae*

**DOI:** 10.1186/s12302-016-0095-4

**Published:** 2016-11-04

**Authors:** Panwad Sillapawattana, Martin C. H. Gruhlke, Andreas Schäffer

**Affiliations:** 1 Institute for Environmental Research (Biology V), RWTH Aachen University, Worringerweg 1, D-52074 Aachen, Germany; 2 Institute for Plant Physiology (Biology III), RWTH Aachen University, Worringerweg 1, D-52074 Aachen, Germany

**Keywords:** Metallothionein, Oxidative stress, Glutathione, Glutathione S-transferase, Ecotoxicological endpoint

## Abstract

**Background:**

Because of their antimicrobial properties, silver nanoparticles (AgNPs) have been widely used and have come into contact with the environment. In the present work, an effect of AgNPs on a standard soil organism, *Folsomia candida,* was studied (in comparison to silver nitrate) focusing on molecular and cellular alterations as ecotoxicological endpoints.

**Results:**

At the molecular level, an up-regulation of metallothionein-containing protein (*MTC*) mRNA in AgNP-treated groups indicated toxic heavy metal stress effects caused by the release of silver ions from AgNPs, which is similar to animal groups treated with silver nitrate. Alteration of the steady-state level of glutathione S-transferase (*GST*) mRNA was detected in animal treated with AgNPs and AgNO_3_. At the cellular level, the relation between GST activity and the size of the glutathione (GSH) was examined. Change of GST activity from different animal groups was not significant, whereas the GSH pool (reduced and oxidized forms) decreased with increasing concentration of AgNPs. In order to obtain direct evidence whether AgNPs cause oxidative stress, treated animals were incubated with the non-fluorescent probe, 2′,7′-dichlorodihydrofluorescein diacetate (DCFH-DA). A fluorescence signal was observed in both AgNPs- and AgNO_3_-treated groups pointing to the production of reactive species (RS). Since RS formation in *F.candida* is difficult to quantify, yeast strain BY4742 (wild-type) and mutants lacking of oxidative stress-related protective enzymes were exploited as a further eukaryote model organism. AgNPs and AgNO_3_ were found to also affect growth of yeast and induced oxidative stress.

**Conclusions:**

An effect of AgNPs on *Collembola* and yeast strains is similar to the one from AgNO_3_. However, AgNPs is less toxic due to the slow release of silver ions. In summary, the toxic effect of AgNPs on *F. candida* is caused by the combination of the release of silver ions from AgNPs and the formation of reactive species.

**Electronic supplementary material:**

The online version of this article (doi:10.1186/s12302-016-0095-4) contains supplementary material, which is available to authorized users.

## Background

Because of their antimicrobial properties, AgNPs have been extensively used in many applications, for example, in textiles, wound dressing, and other medical devices by coating or embedding [1, 2]. AgNPs have unavoidably become part of everyday life and thus are in close contact with human beings and the environment. One possible emerging problem arises from the leakage of AgNPs directly into wastewater [3]. Blaser and co-worker [4] proposed that silver is removed from wastewater at treatment plants and deposited in sewage sludge produced from wastewater treatment. The release of AgNPs to the environment is due to the accumulation in sewage sludge, which is applied as fertilizer in agriculture [5]. At this stage, the re-use of sludge can possibly cause soil and groundwater pollution [4]. Mueller and Nowack reported in their modeling study that the use of sludge as fertilizer released approx. 1 μg nanosilver per kg sludge per year [6]. Some authors speculated that given the limited quantity used, it is unlikely to be a major environmental threat [7]. However, the sewage treatment plant simulation showed that at environmentally relevant concentration more than 90% of AgNPs are bound to sewage sludge and at this concentration, AgNPs absorption to sludge as well as aging in soil have the toxic effects on microorganisms [8].

On the one hand, the practical intent of AgNPs is to disinfect or sterilize a specific type of target organism, in this case toxicity may be interpreted as positive outcome. On the other hand, AgNPs can cause undesired effects on non-target organisms and such toxicity may then pose a potential hazard [9]. Toxicity studies of AgNPs have been widely done during the past decades with animals [10] and mammalian cells [11, 12] including human cells [13, 14]. Lim and co-worker investigated the toxic mechanism of AgNPs in the nematode *Caenorhabditis elegeans*, focusing on the involvement of oxidative stress in toxicity to the reproductive system [10]. The results indicated that exposure to AgNPs led to increased ROS formation, increased expression of PMK-1 p38 mitogen-activated protein kinase (MAPK) and GST enzyme activity and decreased reproduction, which suggested the role of oxidative stress as a significant mechanism of AgNPs-induced reproduction toxicity. Apart from the study in roundworm, Arora et al. explored the effects of nanoparticles on human fibrosarcoma and human carcinoma cells and illustrated that in the presence of AgNPs, the cellular level of glutathione (GSH) was reduced, indicating oxidative stress [15].

The present work investigated the effects of AgNPs on standard test soil organisms *Folsomia candida* (*Collembola*), which have been used as a representative species to study and highlight the effects of chemicals on organisms because of their ease of culture in laboratory and short generation times. Several ecotoxicological tests, including reproductive test [16], metal exposure test [17], and acute test [18] have been introduced in *F. candida*. Although toxicity endpoints (commonly from acute and chronic tests) are routinely used with this species as an input for calculating the Risk Quotient (RQ) in ecotoxicological risk assessment, they provide only a single estimation of environmental risk. The development of more sophisticated methods for conducting ecological risk assessments such as molecular or cellular changes under stress conditions may thus be a promising alternative [19] and can demonstrate a higher sensitivity and response in the initial stages of stress impact [20]. Therefore, in this study, we investigated the effects of AgNPs (in comparison to AgNO_3_) on the test animals focusing at the molecular biological organization level including the expression profiles of target genes as well as at cellular (biochemical) biological organization level by following changes of GST activity and the total GSH amount. Moreover, we studied whether the molecular and cellular changes caused by chemical stress could be used as potential ecotoxicological biomarkers for sub-lethal effects in *F. candida*. Furthermore, reactive species (RS) formation in test animals exposed to AgNPs and AgNO_3_ was also investigated.

The chemical effects were also studied with *Saccharomyces cerevisiae* strains BY4742 and its mutant derivatives by treating them with AgNPs and AgNO_3_ in order to assess the possibility of exploiting yeast as a eukaryotic model organism in ecotoxicological testing, since it shares complex internal cell structure of animals including programmed cell death which can be triggered after application of substances [21]. We also examine whether AgNPs induce oxidative stress in yeast compared to *F. candida*, because oxygen radicals are believed to be a key element of apoptotic execution in yeast [21] and the results should possibly be able to draw the link of molecular toxicity of AgNPs to other higher eukaryotes.

## Methods

### Test material

The experiments were conducted using nanosilver NM-300 K, a reference AgNPs recommended by OECD. The NM-300 K is a well-characterized silver nanoparticles by the European Commission Joint Research Centre (JRC 2011). Briefly, NM-300 K is colloidal silver with a nominal silver concentration of 10% (w/w). NM-300 K is stable over a period of 12 months by UV–VIS analysis. The elution of silver ion from NM-300 K embedded in a poly-acrylic matrix to the physiological phosphate buffer and de-ionized water was 0.01% (w/w) and reached equilibrium after 5 days, and no further increase of silver concentration in the elution media is observed [3].

### Measurement of ionic silver from AgNO_3_

To roughly determine the silver ions in AgNO_3_ solution, 20 mL of 0.1 g/L AgNO_3_ was precipitated into silver chloride using 300 µL of 1 M HCl. The silver chloride was then filtered from the solution through 0.2 µm pore size membrane filter (Schleicher & Schuell, Dassel, Germany) by a vacuum filtration system and weighed. An amount of silver ions was calculated based on the ratio of atomic weights of silver chloride, which is ca. 75.26% by weight.

### *Collembola* culture

*Folsomia candida* (*Collembola*) of European origin was propagated in synchronized laboratory culture according to OECD guideline 2009 [16]. Briefly, Collembola were kept in plastic boxes filled with 50 g of a mixture of calcium sulfate hemihydrate (Sigma-Aldrich, Taufkirchen, Germany) and activated charcoal (Fluka, Buchs, Switzerland) in a ratio of 9:1 (w/w) and cultured at 20 ± 1 °C, a light:dark cycle of 12:12 h. Containers were kept moist at all times ensuring that relative humidity of the air within the containers is close to the saturation. *Collembola* were fed with 0.01 g of granulated dried baker’s yeast (Dr. Oetker, Bielefeld, Germany) placed directly on the surface of the culture substrate in a small heap twice a week. Animals aged 40–60 days were used in all experiment (adapted from OECD guideline to obtain only adults, which are more tolerant to the sucking device during collection than juveniles).

### Exposure of *Collembola* to AgNPs and AgNO_3_

Per assay, 100 synchronized individuals of *F. candida* were placed in a plastic box lined with a culture substrate saturated with 5 mL of 0.01, 0.05, and 0.1 g/L AgNPs (ras material GmbH, Regensburg, Germany) and AgNO_3_ (VEB, Dresden, Germany) for a period of 48 h (7 boxes/one concentration) yielding 1-, 5-, and 10-mg AgNPs/kg culture substrate and 1-, 5-, and 10-mg AgNO_3_/kg culture substrate. Water was used as control. Animals were fed on the first day with dried baker’s yeast (Dr. Oetker, Bielefeld, Germany) and kept in incubator at 20 ± 1 °C, a light:dark cycle of 12:12 h. At the end of the test, animals were fixed in liquid nitrogen and used for RNA extraction and determination of GST activity and GSH pool.

The dose of AgNPs and AgNO_3_ can be calculated as follows: each test vessel contains 50 g of culture substrate. 5 mL of AgNPs and AgNO_3_ (0.01, 0.05, and 0.1 g/L) was added on the surface of the substrate. Exemplarily for the 0.1 g/L AgNO_3_, 5 mL contains 0.5 mg AgNO_3_ added to 50 g of culture substrate corresponding to 10 mg AgNO_3_/kg culture substrate. In the same way, 5 mL of 0.1 g/L AgNPs contains 0.5 mg AgNPs added to 50 g of culture substrate, which is corresponding to 10 mg AgNPs/kg culture substrate.

### qPCR and data analysis

Quantitative real-time PCR was performed on an ABI Prism®7000 sequence detection system (Applied Biosystems, Massachusetts, USA) with three technical replicates per sample. Relative quantification of *GST* and *MTC* expression was carried out using Platinum® CYBR® Green qPCR SuperMix-UDG with ROX (Invitrogen, Carlsbad, USA). Each reaction was done in a final volume of 10 µL containing 2 µL of ten-fold diluted cDNA and 8 µL of master mix. Cycling conditions were kept constant for all assays. Primers for target and reference genes are listed in table below. The characterization of reaction products was done via melting curve analysis and gel electrophoresis to make sure that no formation of primer-dimer exists. Threshold cycle (C_t_) for the gene of interest in both the test samples and control sample were adjusted in relation to a reference gene using the comparative quantification algorithms-ΔC_t_ (Table 1).

**Table 1:** List of primer used for qPCR

Primer name	Sequence (5′–3′)	Target genes	qPCR efficiency (%)	Amplification factor
MTC_forward MTC_reverse	AGCCAATATTTTCGAGTGGAGA CAAGATGCTCGAATAGCAACAGTA	Metallothionein-like containing protein [20]	87.50	1.87
GST_forward GST_reverse	CATCAACATTGCCGACAAAC ACGACAGCCTTGAACTTTGC	Glutathione S-transferase [22]	96.53	1.97
YWHAZ_forward YWHAZ_reverse	TCGCCCTCAACTTTTCCGTT TGCTATCGCTTCATCGAATGC	Tyrosine 3-monooxygenase [23]	88.44	1.88

### Enzyme assay and protein determination

The activity of glutathione S-transferases was determined according to Shen and Chien [24]. Briefly, assays were performed by recording the first 2 min of GS-CDNB conjugation at 25 °C in the reaction mixture consisting of 100 µL of 50 mM GSH (Sigma-Aldrich, Taufkirchen, Germany), 40 µL of 50 mM 1-chloro-2,4-dinitrobenzene (CDNB) (Sigma-Aldrich, Taufkirchen, Germany) dissolved in ethanol, 10 µL of enzyme extract and 0.1 M potassium phosphate buffer, pH 6.5, in a total volume of 2 mL [24]. The reaction was started by the addition of enzyme extract and monitored at 340 nm in Beckman Coulter DU® 800 spectrophotometer (Beckman Coulter, Krefeld, Germany). Activity was calculated using an extinction coefficient of 9.6/mM/cm as described by Habig et al. [25]. Total protein was measured according to the method of Bradford using bovine serum albumin (Bio-Rad, Hercules, USA) as a standard [26]. 1 mL of Bradford reagent (Bio-Rad, Hercules, USA) was added to 20 µL of protein standard and unknown samples. The mixtures were then incubated for at least 5 min at room temperature and measured the absorbance at 595 nm. The standard curves were generated by plotting the absorbance at 595 nm versus their concentration and the unknown sample concentration was determined using the standard curves. Specific activity is defined in unit/mg protein (unit = µmol/min).

### Determination of glutathione in *Collembola*

Pools of *F. candida* from each test condition were ground in liquid nitrogen and 5 volumes (w/v) of sodium-phosphate buffer (143 mM, pH 7.5) containing 10 mg/mL polyvinylpyrrolidone K30 (to hinder browning of the extract resulting in higher yield of GSH during detection). The samples were centrifuged at 20,000*g* for 10 min at 4 °C. The supernatants were collected and determined for total GSH using a standard enzymatic recycling assay in the presence of glutathione reductase (GR) (Sigma-Aldrich, Taufkirchen, Germany) as described by Griffith [27] and Gruhlke et al. [28] with minor modifications. Briefly, GSH was oxidized to GSSG by 5,5′-dithiobis-(2-nitrobenzoic acid) (DTNB) (Sigma-Aldrich, Taufkirchen, Germany) and catalyzed by GR in the presence of NADPH (Carl Roth, Karlsruhe, Germany). The rate of formation of 2-nitro-5-thiobenzoic acid is proportional to the glutathione concentration and was monitored at 412 nm over the period of 2 min and the size of the GSH pool was calculated from a prepared standard curve.

### Reactive species (RS) formation test in *Collembola*

After chemical exposure, test animals were inactivated with nitrogen gas and then fixed in 95% ethanol for a few second. Later, they were washed in phosphate buffer saline (PBS) and finally incubated with PBS buffer containing DCFH-DA (Sigma-Aldrich, Taufkirchen, Germany) for 30 min. After incubation, test animals were washed again in PBS buffer and then placed on a glass slide. The fluorescence can be visualized at 535 nm when excited at 485 nm using Leica DMR microscope (Leica Microsystems Wetzlar GmbH, Wetzlar, Germany). Image analyses were conducted on three individual biological replications.

### Yeast strains and cultivation

Yeast strain BY4742 and mutant derivatives (see table below) were obtained from Euroscarf, University of Frankfurt, Germany. 6 mutants of yeast strain were grown on YPD agar plates (10 g/L yeast extract, 20 g/L peptone, 20 g/L glucose and 20 g/L agar) at 28 °C. Later, the culture was inoculated and allowed to grow overnight in liquid complete synthetic drop-out medium (CSM) containing 7 g/L yeast nitrogen base (ForMedium, Hunstanton, Norfolk, UK), 0.8 g/L complete dropout (Vista, CA, USA), and 40 g/L glucose (Carl Roth, Karlsruhe, Germany) at 28 °C with shaking speed 210 rpm (Table 2).

**Table 2:** List of yeast mutants

Mutant	ORF	Genotype
gsh1Δ	YJL101C	MATα; his3Δ1; leu2Δ0, lys2Δ0, ura3Δ0; YJL101c::kanMX4
gsh2Δ	YOL049W	MATα; his3Δ1; leu2Δ0, lys2Δ0, ura3Δ0; YOL049w::kanMX4
sod1Δ	YJR104C	MATα; his3Δ1; leu2Δ0, lys2Δ0, ura3Δ0; YJR104c::knMX4
sod2Δ	YHR008C	MATα; his3Δ1; leu2Δ0, lys2Δ0, ura3Δ0; YHR008c::knMX4
ctt1Δ	YGR088W	MATα; his3Δ1; leu2Δ0, lys2Δ0, ura3Δ0; YGR088wc::kanMX4

### Yeast toxicity test

Yeast strains were adjusted to an OD_600_ = 0.2 and exposed to AgNPs and AgNO_3_. Controls were treated with CSM medium alone. Tests were conducted in 96-well plates using different concentrations of test substances. Dilution of AgNPs and AgNO_3_ was done using CSM medium. The highest concentration of test substances was transferred to the first row of the plate. Half volume of the test substances in each well from the upper row was transferred to the second row and mixed with the same volume of CSM medium. This manner was repeated with the lower row to obtain all test concentration used. Each test concentration was performed in triplicate. Cultures were incubated for a further 24 h at 28 °C, 210 rpm. Toxic effect of substances was assessed via growth of yeast strains by measurement OD_600_ using plate reader (Berthold Technologies, Bad Wildbad, Germany) and the viability of yeast was assessed via colony formation on CSM medium (please see Additional files 1, 2, 3).

### Reactive species test in yeast

Overnight yeast cultures were adjusted to OD_600_ = 0.5 and treated with 3.125-, 6.25-, 12.5-, 25-, and 50-mg/L AgNPs and AgNO_3_ in 24-well plates and incubated for a further 24 h (28 °C, 210 rpm). 95 µL of test composition in each well was transferred to black flat bottom 96-well plates (repeated 4 times so that 95 µL of test component from the same 24-well plate would be in 4 wells of black 96-well plate). 5 µL of 10 mM DCFH-DA was added to each well and incubated for 30 min. Emission of fluorescence was detected (excitation at 485 nm and emission at 535 nm) using plate reader (Berthold Technologies, Bad Wildbad, Germany).

## Results

### Determination of silver ions released from AgNO_3_

Deionized water was used to get an end concentration of 0.1 g/L AgNO_3_ and the amount of silver ions was observed at 48 h. Silver ions from AgNO_3_ within 48 h were 49.42 ppm ± 5.13, which is approximately equal to 50% (w/w).

### Alteration of steady-state levels of *GST*- and *MTC* mRNA

**Fig. 1:**
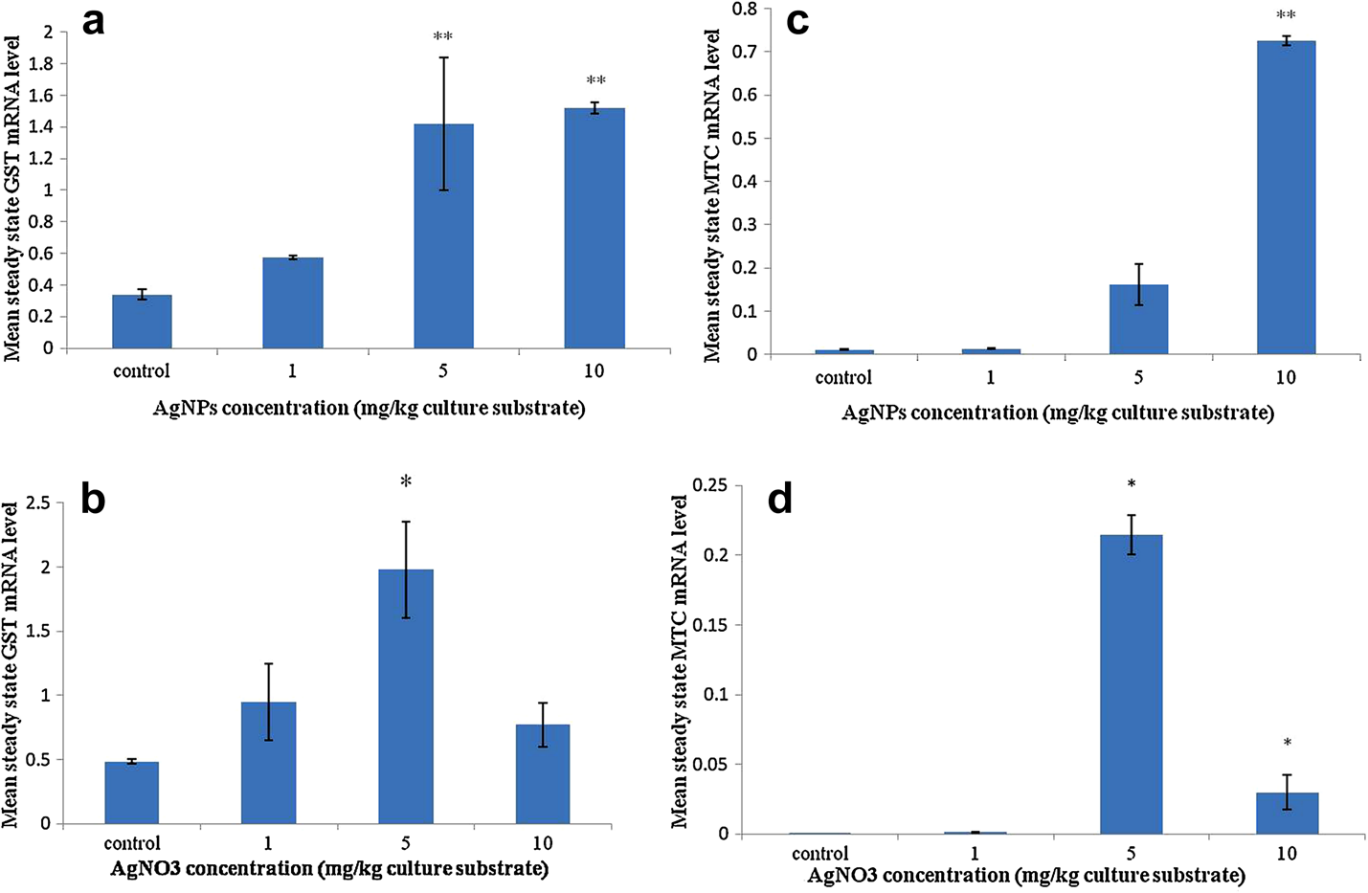
Mean steady-state mRNA level of target gene from *F. candida* treated with AgNPs and AgNO_3_ normalized by reference gene YWHAZ (tyrosine 3-monooxygenase). **a** *GST* mRNA expression of *Collembola* treated with AgNPs; **b** *GST* mRNA expression of *Collembola* treated with AgNO_3_; **c** *MTC* mRNA expression of test animals exposed to AgNPs; **d** *MTC* mRNA expression of test animals exposed to AgNO_3_. Measurements were conducted three times. *Error bars* indicate standard deviations. *Single asterisks* indicate significance at *P* < 0.05; *double asterisks* indicate significance at *P* < 0.01 (using ANOVA with Dunnett’s comparison)

Average threshold cycle (Ct) values obtained from qPCR varied between treatments with different concentrations of test substances and were normalized by reference gene (see “Methods” section). The alteration of the steady-state level of target genes (glutathione S-transferase, *GST* and metallothionein-containing protein, *MTC*) was found in both animals treated with AgNPs and AgNO_3_ (see Fig. 1). An up-regulation of *GST* and *MTC* increased with an increasing concentration of AgNPs. Collembola exposed to 5 and 10 mg/kg AgNPs presented ca. 4 and 4.5 fold up-regulation of *GST* and ca. 14 and 64 fold up-regulation of *MTC* when compared to control, whereas an up-regulation of both target genes could not be detected in *F. candida* treated with the lowest concentration of AgNPs (1 mg/kg). In case of silver salt exposure, compared to control group, *GST*- and *MTC* mRNA steady-stated level was significantly changed in the group treated with 5 mg/kg. Alterations of both target genes declined when animals were treated with the highest salt concentration (10 mg/kg AgNO_3_). At this concentration, the expression level of *GST* could not be detected, whereas the expression of *MTC* was two hundred times higher than that in the control.

### GST activity and GSH amount

**Fig. 2:**
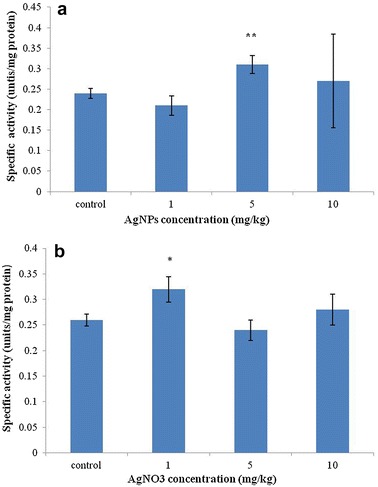
GST activity of protein extracts from *F. candida* treated with AgNPs and AgNO_3_ determined by means of CDNB as a model substrate. **a** GST activity of protein extracts from test animals exposed to different concentrations of AgNPs; **b** GST activity of protein extracts from test animals exposed to different concentrations of AgNO_3_. Measurements were conducted three times. *Error bars* indicate standard deviations. *Single asterisks* indicate significance at *P* < 0.05; *double asterisks* indicate significance at *P* < 0.01 (using ANOVA with Dunnett’s comparison)

**Fig. 3:**
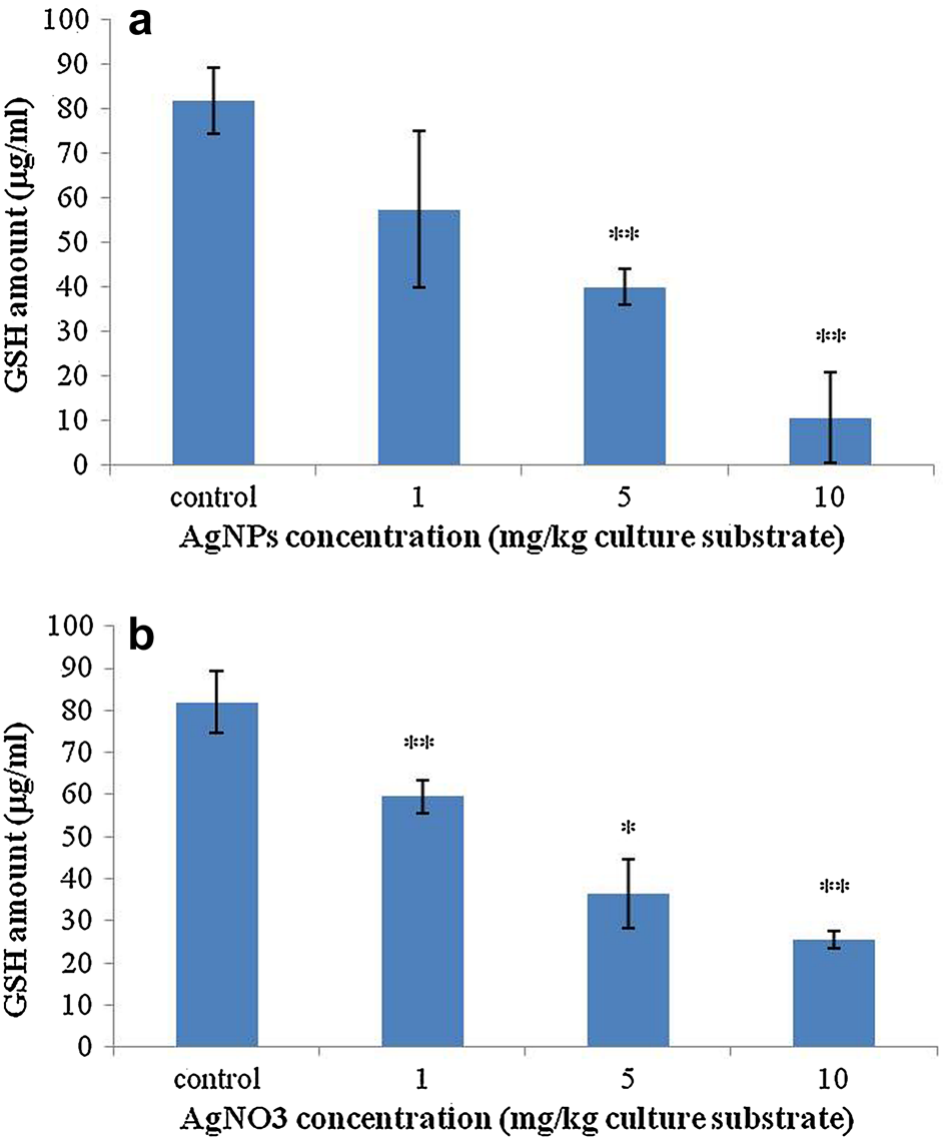
Size of GSH pool from *F. candida* treated with AgNPs and AgNO_3_. **a** Amount of total GSH from test animals treated with AgNPs; **b** pool of GSH from *F. candida* treated with AgNO_3_. The size of the GSH pool decreased with increasing concentration of AgNPs and AgNO_3_. Tests were repeated three times. *Error bars* indicate standard deviations. *Single asterisks* indicate significance at *P* < 0.05; *double asterisks* indicate significance at *P* < 0.01 (using ANOVA with Dunnett’s comparison)

GST was extracted from test animals and activity was measured using 1-chloro-2,4-dinitrobenzene (CDNB) as a model substrate (see Fig. 2). GST activity exhibited a decrease–increase–decrease manner in 1-, 5-, and 10 mg/kg AgNPs-treated animals, respectively. The highest GST activity was found in Collembola exposed to 5 mg/kg AgNPs (0.31 unit/mg protein). In case of AgNO_3_, GST activity presented an increase–decrease–increase manner. The highest activity was detected in animals exposed to 1 mg/kg AgNO_3_ (0.32 unit/mg protein). GSH amount of test animals decreased with increasing concentration of nano-silver and silver salt (see Fig. 3). In the control group, GSH concentration in the extracts of *Collembola* was 81.91 µg/mL. On the other hand, 57.41, 40.07, and 10.71 µg/mL GSH were found in extracts of Collembola treated with 1, 5, and 10 mg/kg AgNPs, respectively. In groups treated with 1, 5, and 10 mg/kg AgNO_3_, GSH concentration in the extracts was 59.56, 36.52, and 25.52 µg/mL, respectively.

### RS formation of *F. candida* exposed to AgNPs and AgNO_3_

**Fig. 4:**
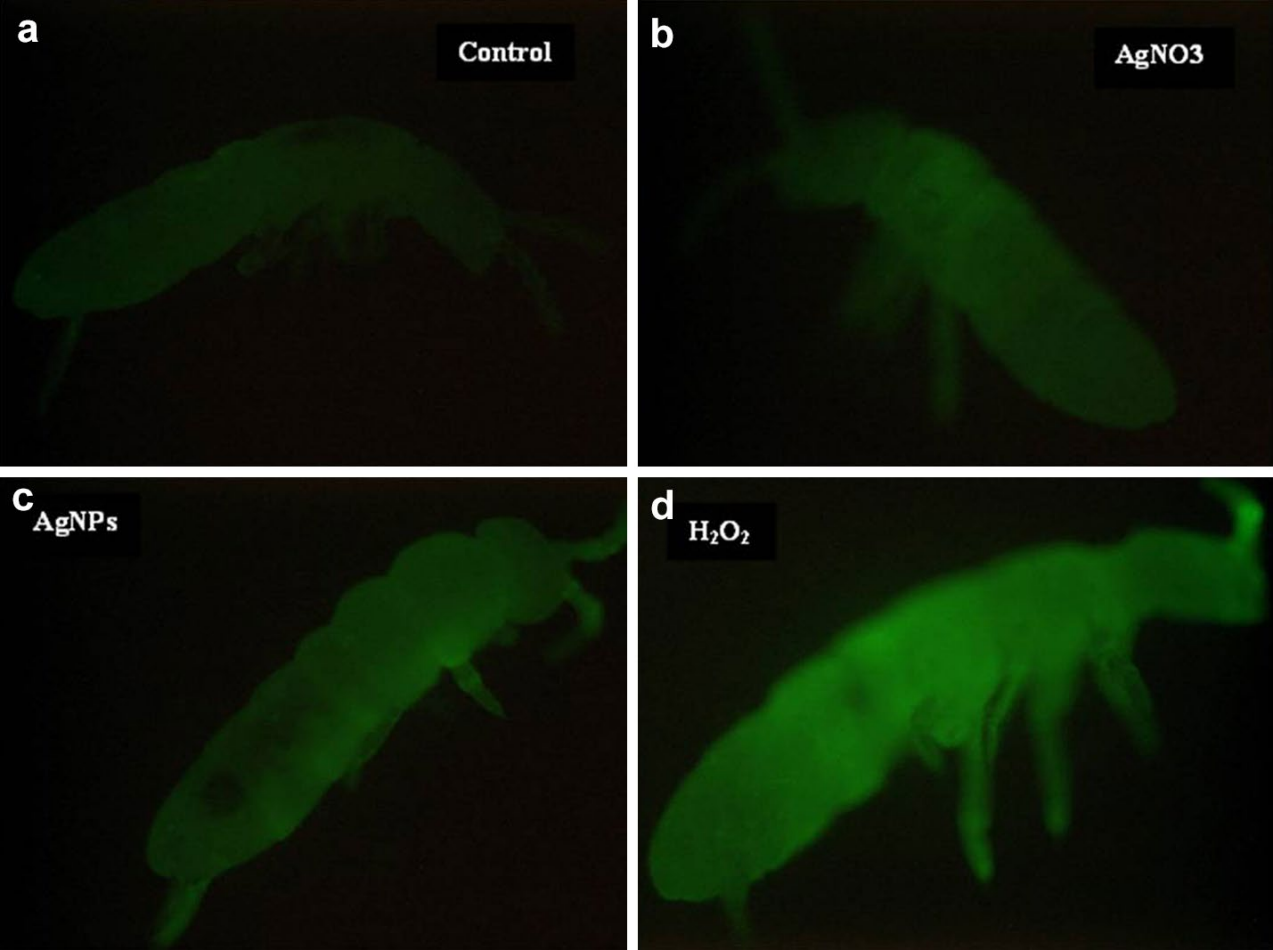
Microscopic image of RS generation illustrate the increase of green fluorescence intensity after DCFH-DA treatment. **a** Control; **b** *Collembola* treated with AgNO_3_; **c** *Collembola* treated with AgNPs; **d** positive control

To obtain the direct evidence whether AgNPs cause oxidative stress to *F. candida*, RS formation was monitored. From Fig. 4, comparing to control, the tendency of increased fluorescence intensity of 2′,7′-dichlorofluorescein (DCF) was observed in both AgNPs- and AgNO_3_-exposed animals. The highest fluorescence intensity was found in positive control (animals were treated with 0.01 M H_2_O_2_), which shows the highest fluorescence intensity compared to the untreated control. Since qualitative method alone was not precisely enough to illustrate the formation of RS, we exploited the quantitative method to measure RS after chemical exposure in yeast.

### Effect of AgNPs and AgNO_3_ to yeast strains

**Fig. 5:**
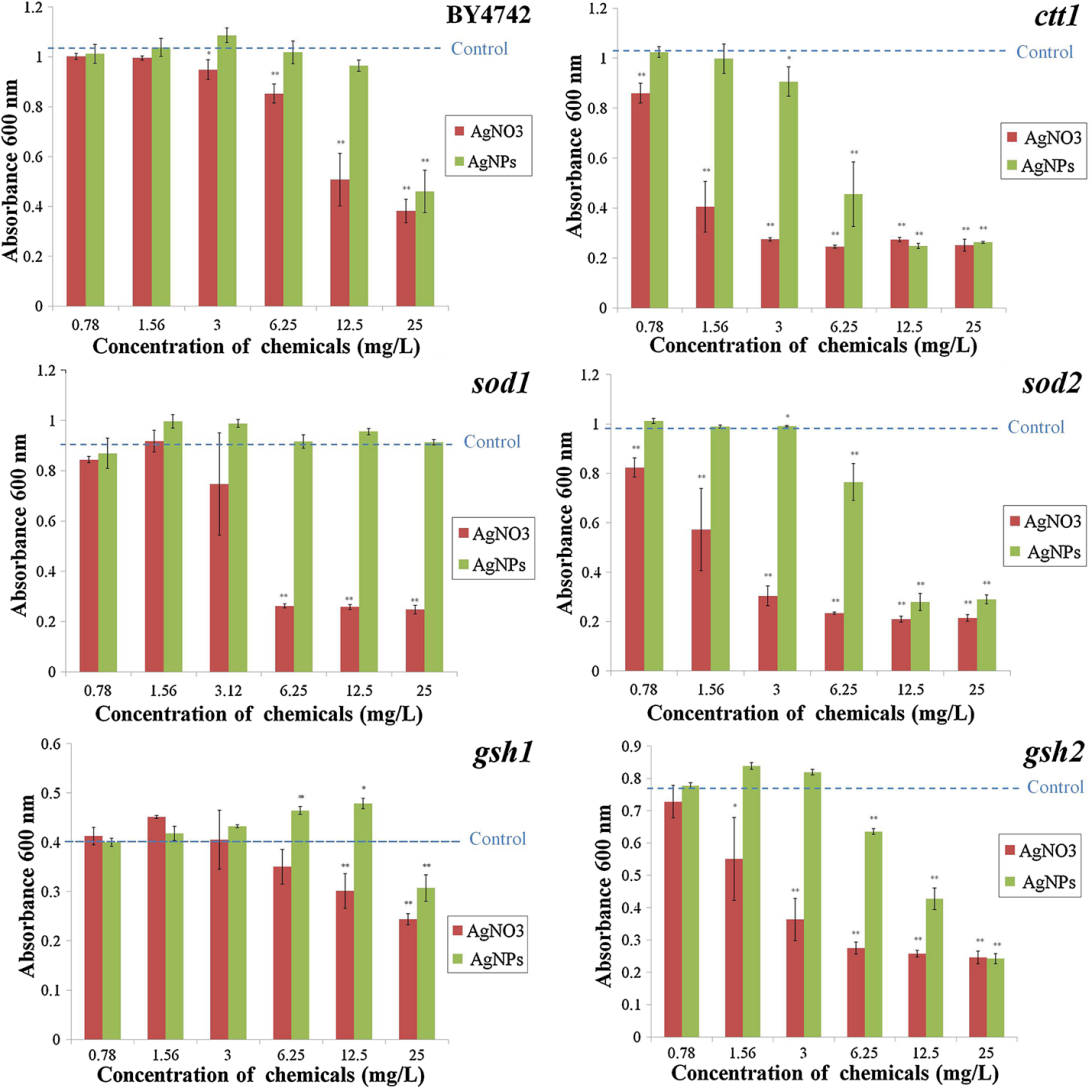
Effect of silver nanoparticles and silver nitrate on BY4742 yeast cells and mutants using simple chemogenetic screening. Besides the wild-type strain BY4742, mutants including *sod1*, *sod2*, *ctt1*, *gsh1*, and *gsh2* have been employed. The *blue dash line* in each graph illustrates the absorbance value of control group (without chemicals). Tests were conducted in triplicate. Significances were calculated by Dunnett’s test compared to control. *Single asterisks* indicate significance at *P* < 0.05; *double asterisks* indicate significance at *P* < 0.01

A toxic effect of AgNPs and AgNO_3_ (compared to control) was investigated with yeast strain BY4742 and its mutant derivatives by measuring absorbance at 600 nm after 24 h of growth (see Fig. 5). For wild-type (BY4742), the absorbance values from control, 0.78, 1.56, and 3 mg/L AgNPs and AgNO_3_, were roughly the same. At the concentration of 6.25 mg/L, the absorbance value of yeast treated with AgNO_3_ sinks to 0.85 and continued to sink when yeast cells were exposed to higher and the highest concentrations. Yeast cells treated with AgNPs obtained similar absorbance values to control using the test concentration below 25 mg/L. In case of chemical exposure in cells lacking the cytosolic catalase gene (*ctt1)*, at 1.56 mg/L AgNO_3_, the optical density (OD) was 0.4 and began to sink when using higher concentration of substance. At and above 3 mg/L AgNO_3_, the OD values were constant. The absorbance values of *ctt1* treated with AgNPs were similar to control when exposed to concentrations 0.78 and 1.56 mg/L. From 3.0 mg/L AgNPs, the absorbance values were lower than control, whereas at 12.5- and 25 mg/L AgNPs, the absorbance values were similar to the mutant treated with AgNO_3_. OD values of cells lacking the cytosolic superoxide dismutase gene (*sod1)* treated with the first two lowest concentrations of AgNO_3_ were alike control and began to sink when treated with 3 mg/L. Afterwards, (from 6.25 to 25 mg/L) the values remained at the same level. Oppositely, all OD values obtained from mutant exposed to different concentrations of AgNPs were similar to control. In case of *sod2* mutant (lacking of the mitochondrial superoxide dismutase gene), absorbance values decreased with increasing concentration of AgNO_3_, while in mutant treated with AgNPs the values were similar to control and began to decline at 6.25 mg/L AgNPs and remained the same when treated with higher concentration. Mutant lacking the gene for GSH synthesis (*gsh1)* exposed to AgNPs and AgNO_3_ obtained roughly the same OD values compared to control, except the last two highest concentrations that the values decreased in mutant treated with AgNO_3_. The OD values obtained from *gsh2* decreased with increasing concentrations of AgNPs and AgNO_3_.

### ROS formation in yeast

**Fig. 6:**
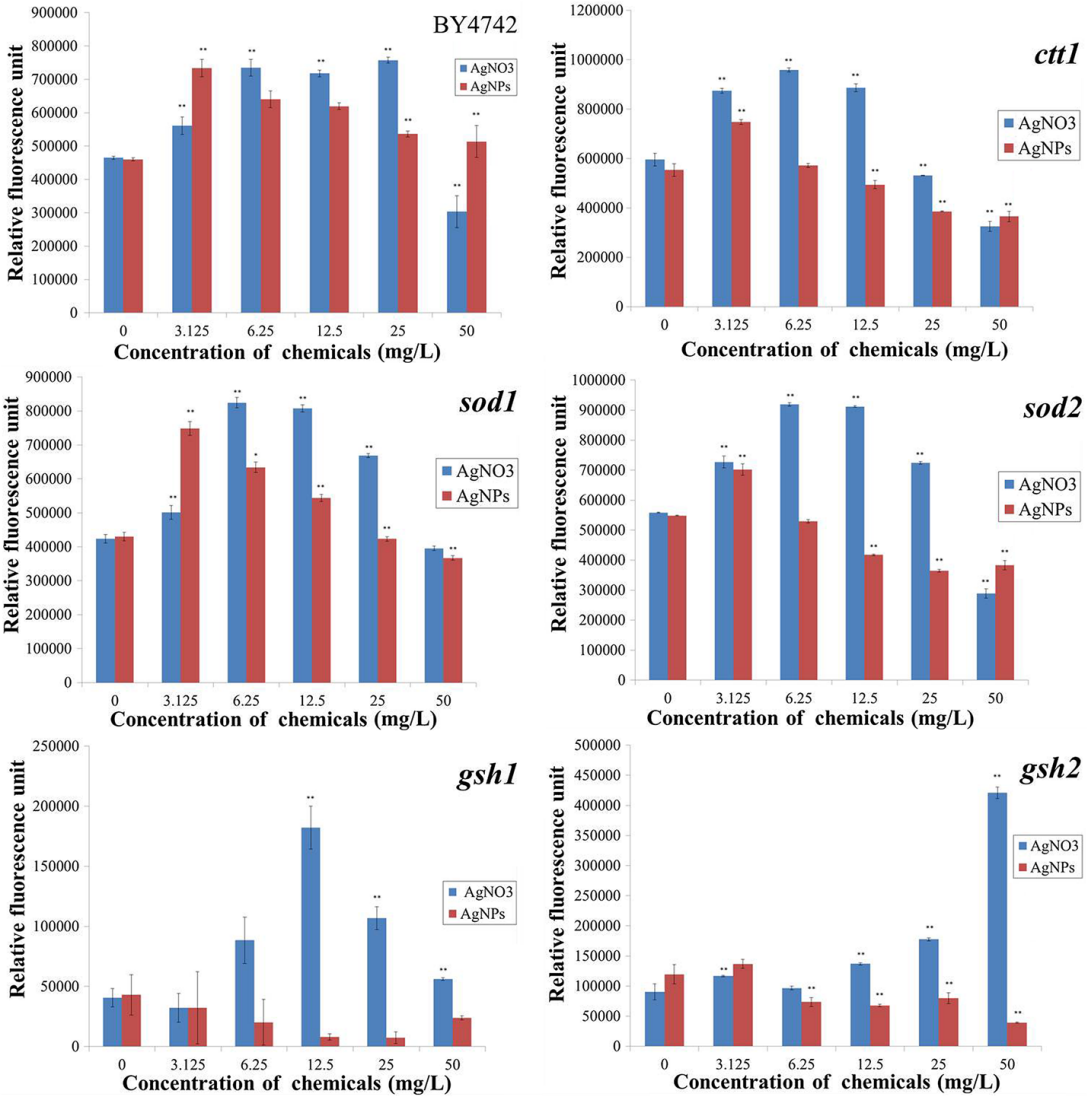
DCFH-DA-based fluorescent yeast assay to determine the formation of RS in response to AgNPs and AgNO_3_. Experiments were done in triplicate. Statistical significances were calculated with ANOVA using Dunnett’s test

From the results obtained from toxicity test in yeast (please see Fig. 5), the concentration above 3 mg/L caused effects. Thus, we chose this concentration for DCFH-DA-based fluorescent assay. The viability of yeast treated with AgNPs and AgNO_3_ was assessed on CSM-agar before conducting ROS test. The colony formation indicated that yeast cells were not dead (please see Additional files 1, 2, 3). From Fig. 6, different concentrations of AgNPs and AgNO_3_ induced ROS formation in yeast distinctly. In all yeast strains treated with AgNPs (except *gsh1*), maximum relative fluorescence values were found in the lowest AgNPs concentration used (3.125 mg/L) and then began to fall when the test concentration increased. Finally, the values remained approximately constant. Conversely, the maximum relative fluorescence value in *gsh1* was obtained in control. In yeast strains exposed to AgNO_3_, the similar pattern of relative fluorescence values was detected in *gsh1*, *ctt1*, *sod1*, and *sod2*. Namely, the highest fluorescence value was detected in 12.5 AgNO_3_-treated strains and consequently dropped as the test concentration increased. In wild-type, the highest relative fluorescence value was observed in the group treated with 25 mg/L AgNO_3_. By contrast, relative fluorescence tended to increase with increasing concentration of AgNO_3_ in *gsh2*.

## Discussion

To answer the hypothesis that the toxic effect of AgNPs is caused by the combination of its nano-size specific properties and the generation of their ions [29], several experiments have been performed in the present study. At the molecular aspect, an alteration of the steady-state level of metallothionein- and glutathione S-transferase mRNA was chosen as ecotoxicological endpoint for AgNPs exposure in *F. candida* (in comparison to silver salt, AgNO_3_). We exploited the expression of *MTC* as biomarker to assess whether the toxic effect of AgNPs is possibly caused by the release of silver ions. An up-regulation of *MTC* was found in both AgNPs- and AgNO_3_ treated groups, which could point to the evidence that silver ions were released from AgNPs and promoted transcription of *MTC* leading to an alteration of the steady state of *MTC* mRNA. Consequently, an up-regulation of this gene could then be detected compared to control animals. An up-regulation of the target gene in AgNO_3_-treated groups was however higher than that in AgNPs-treated groups. This would possibly be due to the higher amount of free silver ions in the silver salt solution than the one released from AgNPs, when using the same test concentration based on the total silver content. The rough determination of Ag ions in AgNO_3_ was approx. 50% (w/w) and the release of Ag ions from AgNPs reported by Klein et al. found to be 0.01% (w/w) [3]. Thus, the amount of silver ions from AgNO_3_ is more than the one released from AgNPs. The slow release of silver ions from AgNPs is indeed an important property of AgNPs which can strongly inhibit the growth of microorganisms such as bacteria, yeast and fungi [2]. AgNPs are sensitive to oxygen and only partially oxidized AgNPs exhibit antimicrobial activity [1]. This property of AgNPs is also related to their size, in which the smaller particles being more active when compared on the basis of equivalent silver mass content [1].

Apart from the change of *MTC* mRNA steady-state level, an expression of a gene believed to encode glutathione S-transferase was also measured. GSTs are involved in detoxification of xenobiotics and reactive oxygen species (ROS). An up-regulation of *GST* was also observed in *Collembola* treated with AgNPs and AgNO_3_. Obviously, *GST* was up-regulated with increasing concentrations of the test substances, except in the group treated with the highest AgNO_3_ concentration in which the steady-state level of *GST* mRNA was nearly the same as in the control group. This is probably because the toxicity threshold was surpassed, as a further indication of toxicity under this condition; less mobility of animals in this group was observed compared to other test groups. The change of *GST* mRNA could possibly indicate oxidative stress which could occur during an exposure of AgNPs and AgNO_3_ as well as an involvement of GST in the removal of reactive oxygen species.

At the molecular organization level, an up-regulation of target genes (*GST* and *MT*) from *F. candida* exposed to AgNPs provides evidence of the ability of the test animals to maintain homeostasis in a changing environment. This consequently drives organisms to the boundaries of their ecological niche and induces stress responses [30]. Accordingly, Nota et al. reported that the identified biological processes, which are involved in environmental toxicity, can function as new genomic endpoints [31]. This demonstration consequently points to the possibility of employing gene expression to examine the effects of chemicals on test organisms in toxicogenomic studies [32]. Since the signal transduction pathways, in which stress affects gene transcription, is recently well-understood, this approach might suggest that the diagnosis of environmental quality by transcriptional profiling is possible [33]. Nevertheless, when measuring steady-state levels of mRNA, one should keep in mind not to overestimate chemical toxicity on other organization levels (cells, individuals and population), because gene expression alone can sometimes be a poor predictor of protein abundance [34].

At the cellular level, change of GST activity was not significant in both animal groups and no correlation between enzyme activity and the test concentration used could be observed. However, the enzyme levels are often increased by exposure of the animals to xenobiotics, because the binding of antioxidant response element (ARE) and Nrf1 and Nrf2 proteins allows increased transcription of gene encoding GSTs [35]. This is possible because the used concentrations were not either fine enough or more chemical concentration in between should be tested in order to obtain the tendency of enzyme activity.

Since GST catalyzes GSH conjugation reactions, the pool of GSH was determined to study the relation between GST activity and GSH amount during chemical exposure. It is obvious that the amount of total GSH (reduced and oxidized forms) decreased with an elevated concentration of both silver salt and nanosilver. This would be consistent with the study of *F. candida* exposed to insecticide imidacloprid that the highest amount of GSH was found in control animals and GSH amount decreased when pesticide concentration rose [22]. In the same way, Arora et al. showed that AgNPs exposure reduced the cellular level of GSH in human fibrosarcoma and human carcinoma cells, which indicates an involvement of oxidative stress [15]. Apparently, GSH was used during chemical exposure for the purpose of detoxification, which reflects a vital role of GSH as an antioxidant and as the major thiol-disulfide redox buffer of the cells by producing disulfide bonds from cysteine thiols of GSH and forms oxidized GSH (glutathione disulfide abbreviated as GSSG) [19]. The relation between the concentration of GSH and the resistance of oxidative stress could be illustrated by Nernst equation. The reduction potential of the GSSG/2GSH half-cell (*E*_hc_) can be identified as follows [36]:

1 $$ \begin{aligned}E_{\text{hc}}& = - 240 - \left( {{{59.1} \mathord{\left/ {\vphantom {{59.1} 2}} \right. \kern-0pt} 2}} \right)\log ( {{{ [ {\text{GSH}} ]^{2} } / { [ {\text{GSSG}}]}}} ){\rm mV}\\ &\quad\quad{\text{at 25}}\;{^\circ }{\text{C, pH 7}} . 0. { }\end{aligned} $$

From the Nernst equation, the concentration of GSH does not only influence the half-cell reduction potential (*E*_hc_), but also the buffering capacity of the cellular redox environment. The redox environment of a cell alters throughout its life cycle and moves cells through different biological stages. During proliferation, *E*_hc_ for the GSSG/2GSH redox couple is at the lowest level, but increase when cells undergo differentiation. Beyond a toxic threshold of *E*_hc_, death signals are then activated and apoptosis is initiated. Very high values of *E*_hc_ (GSH) are caused by severe oxidative stress, which will initiate necrosis as a path to cell death [36]. This is a good example why cell with higher [GSH] might be more resistant to oxidative stress than cell contains lower one. The decrease of GSH amount of treated animals also could point to an involvement of oxidative stress upon chemical exposure, as xenobiotic disturb the redox status in organisms and cause an imbalance [22]. From the fact that oxidative stress is involved in the initial stages of stress responses, and is responsive at low doses of chemical exposure, it could address a cause–effect relationship [37]. Therefore, it may be possible to employ GSH level as an oxidative stress biomarker in ecotoxicological studies within environmental risk assessments.

According to Mendes and co-worker, the amount of MT in *F. candida* treated with AgNPs and AgNO_3_ was increased at 48-h exposure and the amount of MT treated with the latter was higher than with the former one [38]. Furthermore, at population level, a dose–response effect of AgNPs is lower than that of AgNO_3_ [38]. The increased amount of MT at cellular level is directly correlated to an up-regulated *MTC* mRNA and might confirm the toxic effect to *F. candida* via silver ions released from AgNPs.

The increased fluorescence of *F. candida* treated with AgNPs and AgNO_3_ could indicate the formation of RS and may support the fact that the amount of GSH decreased because it was exploited in detoxifying the metabolites from oxidative reactions. Indeed, the generation of ROS is one of the most common toxic mechanisms during AgNPs exposure in bacteria [9], roundworms [10], and human cells [14]. However, RS formation in *F. candida* is difficult to quantify; eukaryote model organisms were employed as more informative analytical alternative. A number of yeast mutants, which are deleted in genes encoding oxidative stress-related enzymes such as superoxide dismutase (SOD), glutathione synthetase (GSH), and glutathione reductase (GR), were exposed to AgNPs and AgNO_3_. Such mutants would indicate towards a possible link between the missing enzyme and its cellular roles as well as the biochemical action of substances tested. In fact, for the enzymes of glutathione metabolism, the yeast genome has only one gene of particular enzyme, whereas for superoxide dismutases, the yeast genome contains one gene for a cytosolic (*SOD1*) and a mitochondrial (*SOD2*) enzyme, respectively [39]. Because of this simple genetic accessibility of particular biochemical key players as well as an easy experimental handling of yeast, this organism could serve as a reasonable and advantageous system for chemogenetic screening [40]. Interestingly, the chemogenetic analysis showed a significant toxicity of AgNPs and AgNO_3_ in yeast cells. However, as the distinct toxicity of AgNPs and AgNO_3_ is concerned, it is possible that yeast cells are either more sensitive to AgNO_3_ or can handle better with AgNPs. AgNPs are not toxic to strain lacking of copper-zink superoxide dismutase (*sod1*) and shown less toxic effects toward the *gsh1* mutant. AgNO_3_, oppositely, was toxic to all strains. The possible involvement of reactive oxygen species was confirmed by assessing the oxidation of the non-fluorescent probe DCFH-DA [41]. The fluorescence caused by the AgNPs and AgNO_3_ exposure relative to that in control organisms was measured. The formation of ROS derived from AgNPs and AgNO3 exposure was detected in all mutant strains, which was similar to the data obtained from the former experiment conducted with *F. candida*.

Since the limitation in quantifying RS level in *F. candida* could be circumvented by exploiting yeast and all mutants. The yeast-based screening is thus an informative alternative, and could potentially be used as a model organism for exposure to xenobiotics within an ecotoxicological context [42], particularly in the application of oxidative stress-related mechanism, because yeast possesses a cellular redox chemistry which is comparable to higher eukaryotes albeit in a less complex manner [43] or even to understand the correlation between oxidative stress and disease development [44].

## Conclusions

Toxic effects of AgNPs on *F. candida* may be caused either by the combination of the release of silver ions and RS formation, or by the release of silver ions, which consequently leads to formation of RS. Furthermore, exposure with AgNPs generated significant levels of ROS in eukaryote model organisms. In comparison, silver nitrate and nanosilver have similar effect on both molecular and cellular level of test animals. However, the former induced a higher degree of *MTC* up-regulation, which is likely due to a higher amount of free silver ions within the test environment. One should further determine the exact amount of silver ions with the suitable equipment.

Our results provide a basis for testing the impact of chemicals in *Collembola* on a sub-lethal molecular and cellular level. However, the toxicity of AgNPs to *Collembola* using an artificial substrate instead of soil must be considered with care because in soil environmental processes such as leaching and sorption may lead to different results.

## Additional files

**Additional file 1.** Standard curves.

**Additional file 2.** Yeast colony formation.

**Additional file 3.** Fluorescent intensity relative to control.

## Abbreviations

AgNPs: silver nanoparticles
CDNB: 1-chloro-2,4-dinitrobenzene
DCFH-DA: 2′,7′-dichlorodihydrofluorescein diacetate
DTNB: 5,5′-dithiobis-(2-nitrobenzoic acid)
GR: glutathione reductase
GSH: glutathione
GSSG: glutathione disulfide
GST: glutathione S-transferase
MTC: methallothionein-like containing protein
qPCR: quantitative polymerase chain reaction
ROS: reactive oxygen species
YWHAZ: tyrosine 3-monooxygenase

## References

[1] Lok C, Ho C, Chen R, He Q, Yu W, Sun H, Tam PK, Chiu J, Che C (2007). Silver nanoparticles: partial oxidation and antibacterial activities. J Biol Inorg Chem.

[2] Rai M, Yadav A, Gade A (2009). Silver nanoparticles as a new generation of antimicrobials. Biotechnol Adv.

[3] Klein CL, Comero S, Stahlmecke B, Romazanov J, Kuhlbusch TAJ, Doren EV, Temmerman P-JD, Mast J, Wick P, Krug H, Locoro G, Hund-Rinke K, Kördel W, Friedrichs S, Maier G, Werner J, Lingsinger T, Gawlik BM (2011) NM-series of representative manufactured nanomaterials NM-300 silver characterization, stability, homogeneity. JRC scientific and technical reports 60709

[4] Blaser SA, Scheringer M, MacLeod M, Hungerbühler K (2008). Estimation of cumulative aquatic exposure and risk due to silver: contribution of nano functionalized plastics and textiles. Sci Total Environ.

[5] Wiechmann B, Dienemann C, Kabbe C, Brandt S, Vogel I, Roskoch A (2012) Klärschlammentsorgung in der Bundesrepublik Deutschland. Umweltbundesamt

[6] MuellerNC Nowack B (2008). Exposure modeling of engineered nanoparticles in the environment. Envir Sci Tech.

[7] Valsami-Jones E, Lynch I (2015). How safe are nanomaterials?. Science.

[8] Schlich K, Klawonn T, Terytze K, Hund-Rinke K (2013). Hazard assessment of a silver nanoparticle in soil applied via sewage sludge. Environ Sci Eur.

[9] Marambio-Jones C, Hoek EMV (2010). A review of the antibacterial effects of silver nanoparticles and potential implications for human health and the environment. J Nanopart Res.

[10] Lim D, Roh J, Eom H, Choi J, Hyun JW, Choi J (2012). Oxidative stress-related PMK-1 p38 MAPK activation as a mechanism for toxicity or silver nanoparticles to reproduction in the nematode *Caenorhabditis elegans*. Environ Toxicol Chem.

[11] Awasthi KK, Awasthi A, Verma R, Kumar N, Roy P, Awasthi K, John P (2015). Cytotoxic, genotoxicity and alteration of cellular antioxidant enzymes in silver nanoparticles exposed CHO cells. Royal Soc Chem.

[12] Hsin Y, Chena C, Huang S, Shih T, Lai P, Chueh P (2008). The apoptotic effect of nanosilver is mediatedby a ROS-and JNK-dependent mechanism involving the mitochondrial pathway in NIH3T3 cells. Toxicol Lett.

[13] Piao MJ, Kang KA, Lee IK, Kim HS, Kim S, Choi JY, Choi J, Hyun JW (2011). Silver nanoparticles induce oxidative cell damage in human lever cells through inhibition of reduced glutathione and induction of mitochondria-involved apoptosis. Toxicol Lett.

[14] Miura N, Shinohara Y (2009). Cytotoxic effect and apoptosis induction by silver nanoparticles in HeLa cells. Biochem Bioph Res Co.

[15] Arora S, Jain J, Rajwade J, Paknikar K (2008). Cellular responses induces by silver nanoparticles: in vitro study. Toxicol Lett.

[16] OECD (2009). Guidelines for testing chemicals no. 232: Collembolan reproduction test in soil.

[17] Fountain MT, Hopkin SP (2001). Continuous monitoring of *Folsomia candida* (Insecta: *Collembola*) in a metal exposure test. Ecotox Environ Safe.

[18] Wolf-Roskosch F (1983). Standardisierte Testverfahrung zur Prüfung der akuten Toxizität von Umweltchemikalien an Springschwänzen (*Collembola*) unter besonderer Berücksichtigung von *Folsomia candida*. UBA Texte 27/83 Chemikaliengesetz, Heft3.

[19] Stenersen J (2004). Chemical pesticide: mode of action and toxicity.

[20] Nakamori T, Fujimori A, Kinoshita K, Ban-nai T, Kubota Y, Yoshida S (2010). mRNA expression of a cadmium-responsive gene is a sensitive biomarker of cadmium exposure in the soil collembolan *Folsomia candida*. Environ Pollut.

[21] Madeo F, Kerker E, Wissing S, Jungwirth H, Eisenberg T, Fröhlich K (2004). Apoptosis in yeast. Curr Opin Microbiol.

[22] Sillapawattana P, Schäffer A (2016). Effect of imidacloprid on detoxifying enzyme glutathione S-transferase on *Folsomia candida* (*Collembola*). Environ Sci Pollut R.

[23] de Boer ME, de Boer TE, Marien J, Timmermans MJTN, Nota B, van Straalen NM, Ellers J, Roelofs D (2009). Reference genes for QRT-PCR tested under various stress conditions in *Folsomia candida* and *Orchesella cincta* (Insecta, *Collembola*). BMC Mol Biol.

[24] Shen S, Chien C (2003). Induction of glutathione S-transferases activities in *Drosophila melanogaster* exposed to phenol. Arch Insect Biochem.

[25] Habig WH, Pabst MJ, Jakoby WB (1974). Glutathione S-transferases: the first enzymatic step in mercapturic acid formation. J Biol Chem.

[26] Bradford MM (1976). A rapid and sensitive method for the quantitation of microgram quantities of protein utilizing the principle of protein-dye binding. Anal Biochem.

[27] Griffith OW (1980). Detemination of glutathione and glutathione disulfide using glutathione reductase and 2-vinylpyridine. Anal Biochem.

[28] Gruhlke MCH, Portz D, Stitz M, Anwar A, Schneider T, Jacob C, Schlaich NL, Slusarenko AJ (2010). Allicin disrupts the cell’s electrochemical potential and induces apoptosis in yeast. Free Radical Bio Med.

[29] Wijnhoven SWP, Peijnenburg WJGM, Heberts CA, Hagens WI, Oomen AG, Heugens EHW, Roszek B, Bisschops J, Gosens I, Meent DVD, Dekkerks S, Dejong WH, Zijverden MV, Sips AJAM, Geertsma RE (2009). Nano silver-A review of available data and knowledge gaps in human and environmental risk assessment. Nanotoxicology.

[30] Burnaford JL (2004). Habitat modification and refuge from sublethal stress drive a marine plant-herbivore association. Ecology.

[31] Nota B, Timmermans MJTN, Franken O, Montagne-Wajer K, Marien J, De Boer ME, De Boer TE, Ylstra B, Van Straalen NM, Roelofs D (2008). Gene expression analysis of *Collembola* in cadmium containing soil. Environ Sci Technol.

[32] Littieri T (2006). Recent applications of DNA microarry technology to toxicology and ecotoxicology. Environ Health Persp.

[33] Roelofs D, Aarts MGM, Schat H, Van Straalen NM (2007). Functional ecological genimics to demonstrate general and specific responses to abiotic stress. Funct Ecol.

[34] Vogel C, de Sousa Abreu R, Ko D, Le S, Shapiro BA, Burns SC, Sandhu D, Boutz DA, Marcotte EM, Penalva LO (2010). Sequence signatures and mRNA concentration can explain two-thirds of protein abundance variation in a human cell line. Mol Syst Biol.

[35] ItohK (2004). Molecular mechanism activating Nrf2-Keap1 pathway in regulation of adaptive response to electrophiles. Free Rad BiolMed.

[36] Schafer FQ, Buettner GR (2001). Redox environment of the cell as viewed through the redox state of the glutathione disulfide/glutathione couple. Free Radical Bio Med.

[37] Mugoni V, Camporeale A, Santoro MM (2014). Analysis of oxidative stress in zebrafish embryos. J Vis Exp.

[38] Mendes LA, Maria VL, Scott-Fordmand JJ, Amorim MJB (2015). Ag nanoparticles (Ag NM300 K) in the terrestrial environment: effects at population and cellular level in *Folsomia candida* (*Collembola*). Int J Environ Res Public Health.

[39] Doering M, Diesel B, Gruhlke MCH, Viswanathan UM, Mániková D, Chovanec M, Burkholz T, Slusarenko AJ, Kiemer AK, Jacob C (2012). Selenium- and tellurium-containing redox modulators with distinct activity against macrophages: possible implications for the treatment of inflammatory diseases. Tetrahedron.

[40] Auerbach D, Arnoldo A, Bogdan B, Fetchko M, Stagljar I (2005). Drug discovery using yeast as a model system: a functional genomic and proteomic view. Curr Proteom.

[41] Rosenkranz AR, Schmaldienst S, Stulmeier KM, Chen W, Knapp W, Zlabinger GJ (1992). A microplate assay for the detection of oxidative products using 2′, 7′-dichlorofluorescin-diacetate. J Immunol Methods.

[42] Gasch AP, Hohmann S, Mager WH (2003). The environmental stress response: a common yeast response to diverse environmental stresses. Yeast stress responses.

[43] de la Torre-Ruiz MA, Pujol N, Sundaran V (2015). Coping with oxidative stress. The yeast model. Curr Drug Targets.

[44] Breitenbach M, Ralser M, Perrone GG, Iglseder B, Rinnerthaler M, Dawes IW (2013). Oxidative stress and neurodegeneration: the yeast model system. Front Biosci (Landmark edition).

