# Supplementary material for: Effect of silver nanoparticles on the standard soil arthropod Folsomia candida (Collembola) and the eukaryote model organism Saccharomyces cerevisiae
Source: Environ Sci Eur. 2016 Nov 4;28(1):27. doi: 10.1186/s12302-016-0095-4 (PMC5097105; doi:10.1186/s12302-016-0095-4)

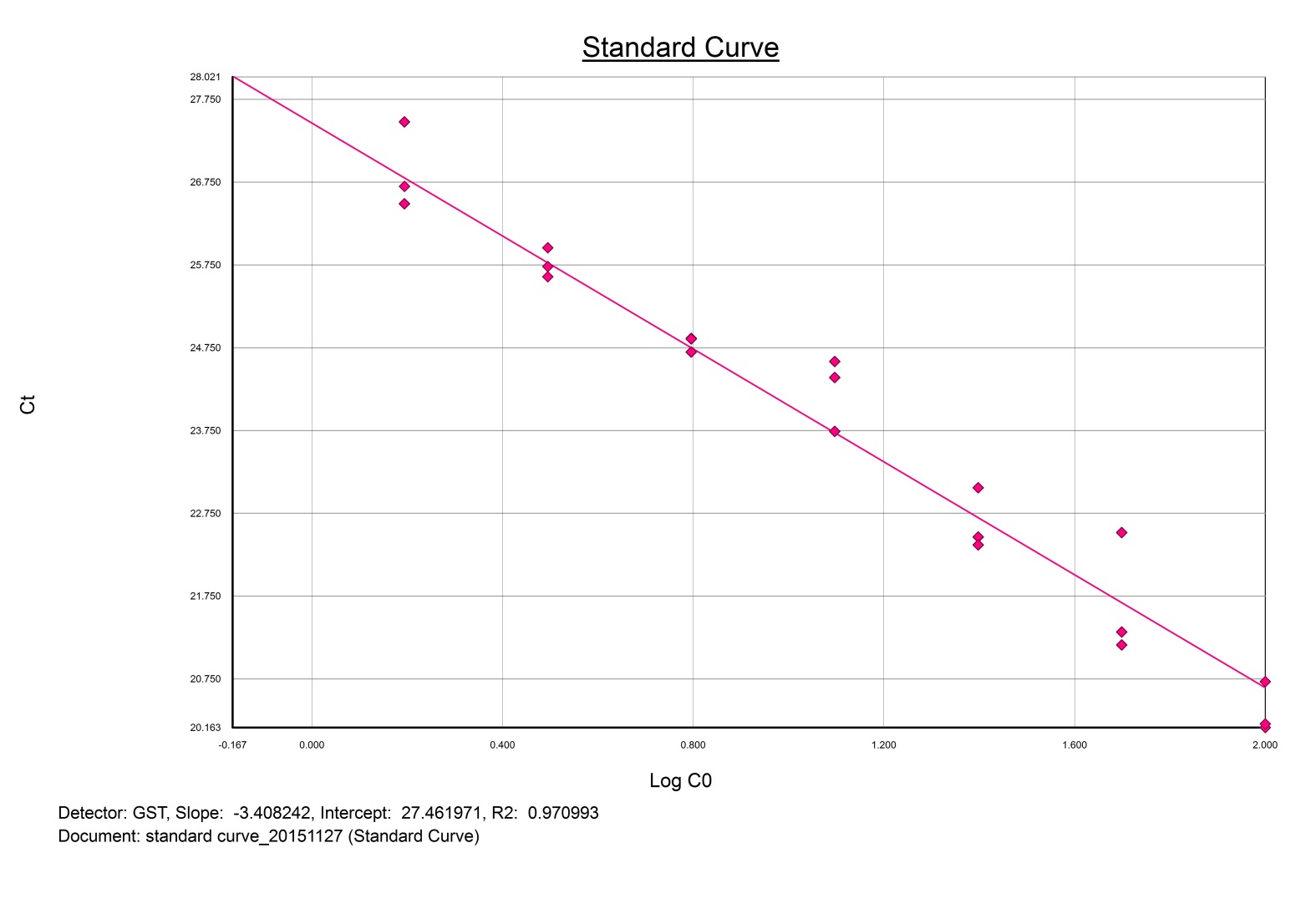
Standard curves obtained from *F. candida* treated with 5 mg/kg culture substrate as cDNA template in the presence of primer specific for *GST*, *YWHAZ* and *MTC*. The measured Ct values are plotted against the log of copy number of the template to establish the standard curve that permits the calculation of the efficiency from the slopes and r^2^ values.


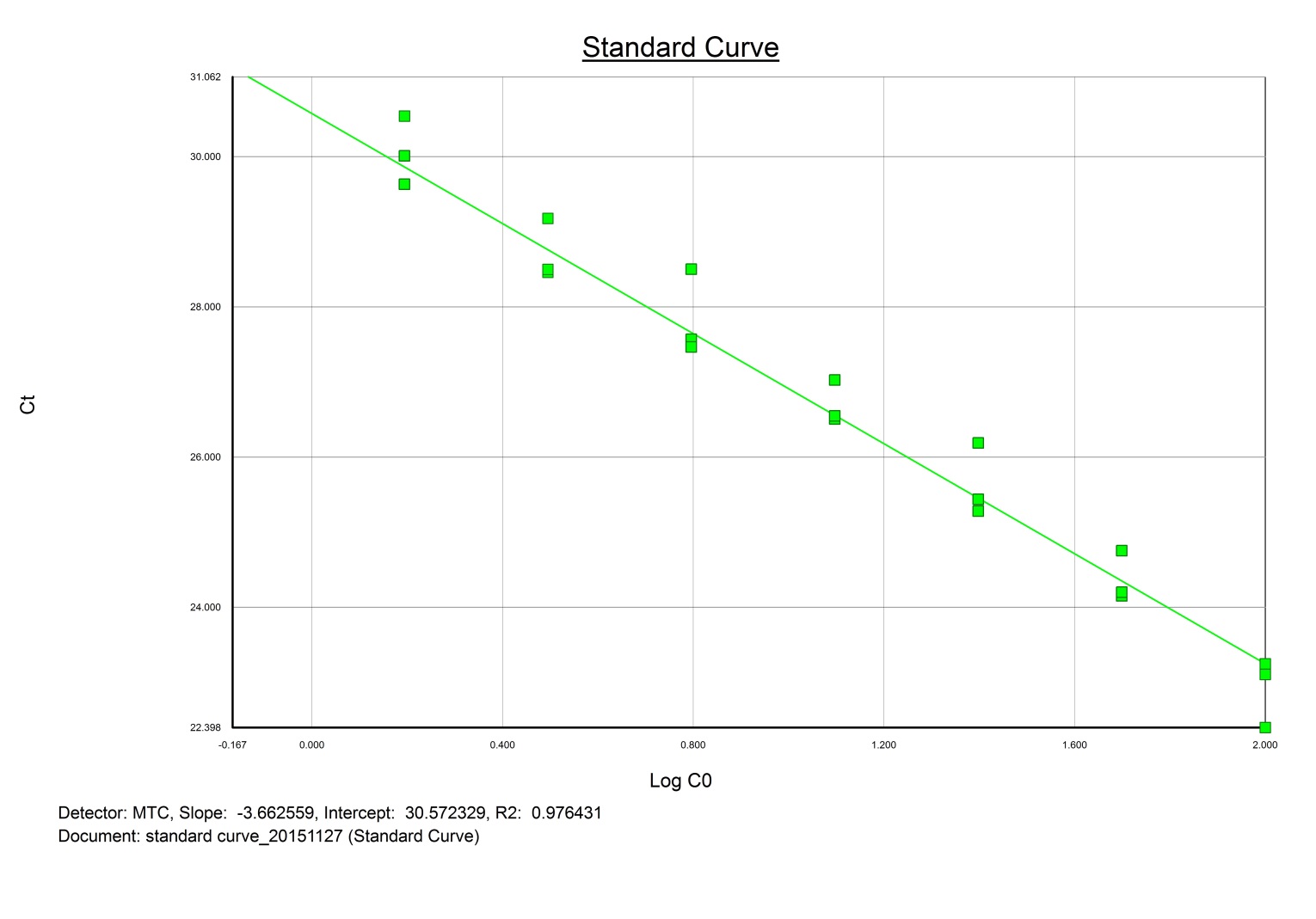

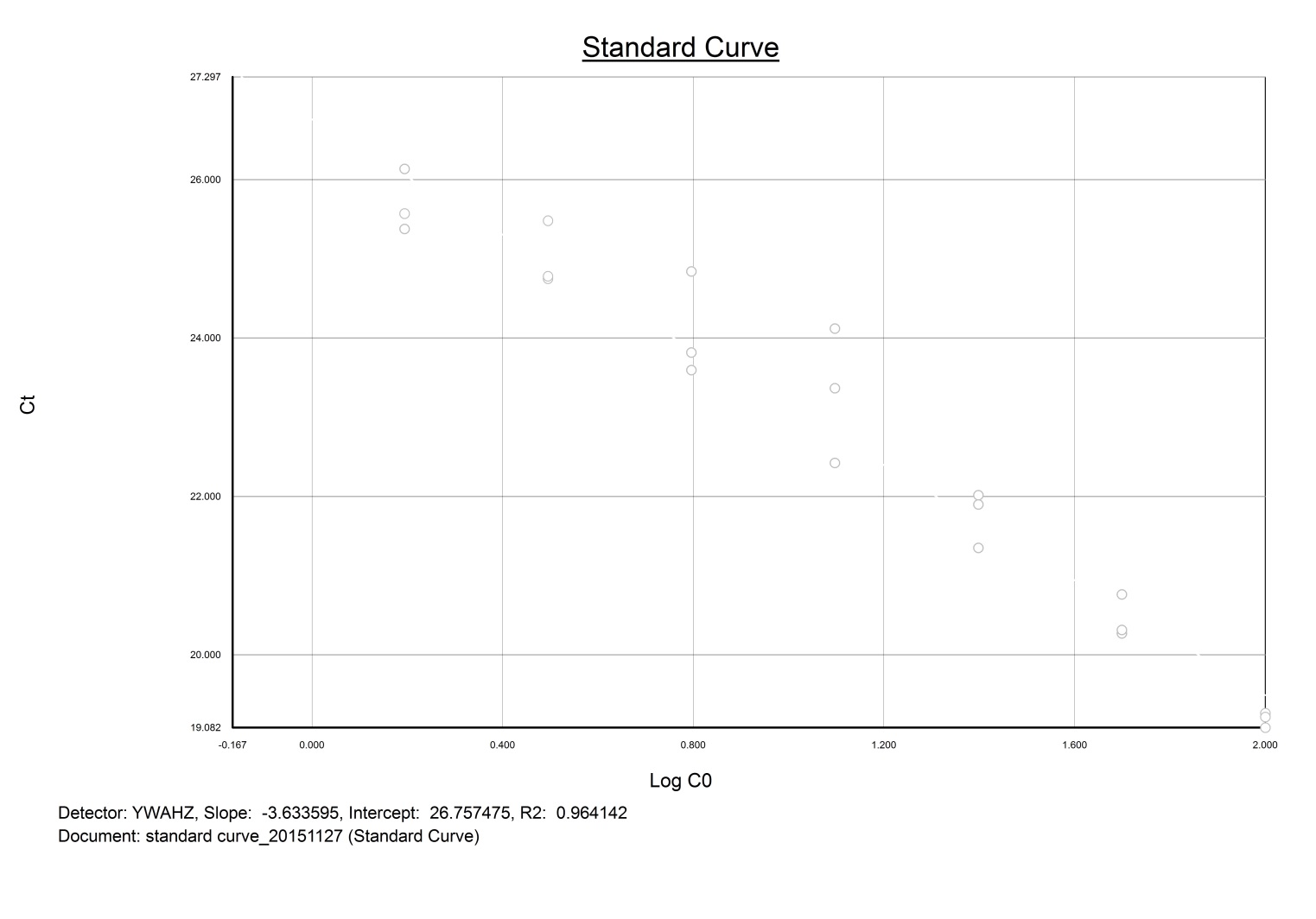

Supplement: Supplementary file 1 — Additional file 1. Standard curves. [file 12302_2016_95_MOESM1_ESM.docx]
