# Supplementary material for: Effect of silver nanoparticles on the standard soil arthropod Folsomia candida (Collembola) and the eukaryote model organism Saccharomyces cerevisiae
Source: Environ Sci Eur. 2016 Nov 4;28(1):27. doi: 10.1186/s12302-016-0095-4 (PMC5097105; doi:10.1186/s12302-016-0095-4)
Supplement: Supplementary file 2 — Additional file 2. Yeast colony formation. [file 12302_2016_95_MOESM2_ESM.docx]

Three figures below show the colony formation of wild-type yeast and mutants on CSM agar after exposed to different concentration of AgNPs and AgNO_3_. The number in front of each row states for the test concentration (mg/L).

AgNPs

AgNO_3_3

**Fig. S1 Colony formation of yeast strain *sod1* and *sod2* treated with different concentration of AgNPs and AgNO_3_.**

**Fig. S2 Colony formation of wild-type and mutants (*gsh1, gsh2, ctt1*) exposed to AgNO_3_.**

**Fig. S3 Colony formation of wild-type and mutants (*gsh1, gsh2, ctt1*) after AgNPs exposure.**
