# Supplementary material for: Effect of silver nanoparticles on the standard soil arthropod Folsomia candida (Collembola) and the eukaryote model organism Saccharomyces cerevisiae
Source: Environ Sci Eur. 2016 Nov 4;28(1):27. doi: 10.1186/s12302-016-0095-4 (PMC5097105; doi:10.1186/s12302-016-0095-4)
Supplement: Supplementary file 3 — Additional file 3. Fluorescent intensity relative to control. [file 12302_2016_95_MOESM3_ESM.docx]

Six figures below illustrate fluorescent intensity relative to untreated control from each mutant. Experiments were done in triplicate. Statistical significance was calculated with ANOVA using Dunnett’s test compared to control. (Single asterisks indicate significance at P < 0.05; double asterisks indicate significance at P < 0.01).

*
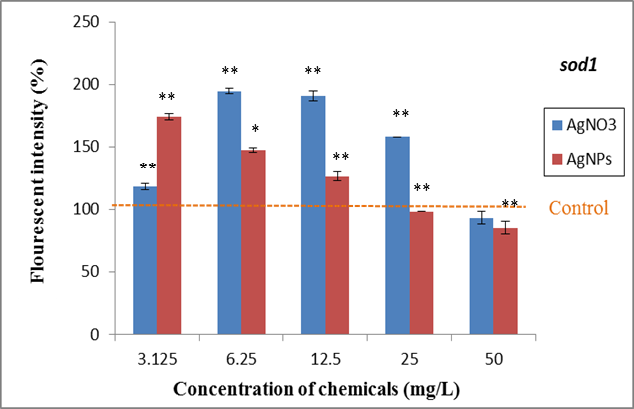
*
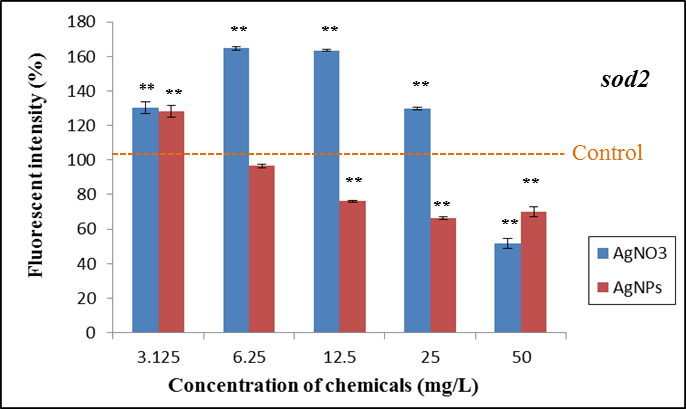

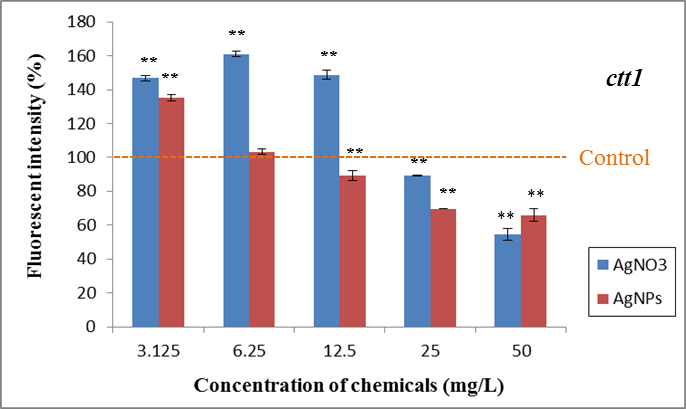

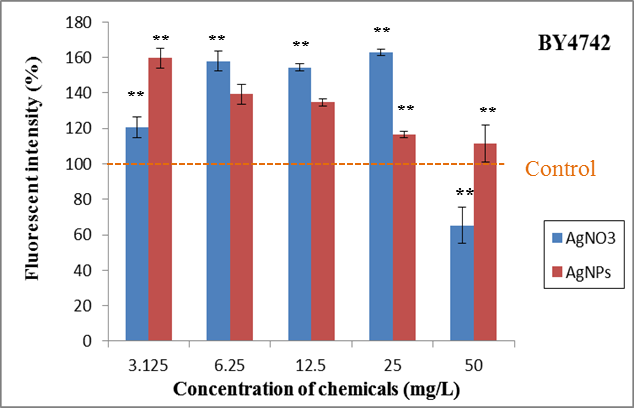


*
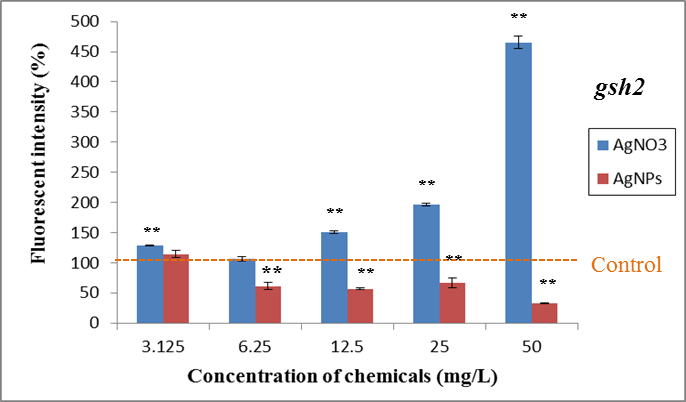

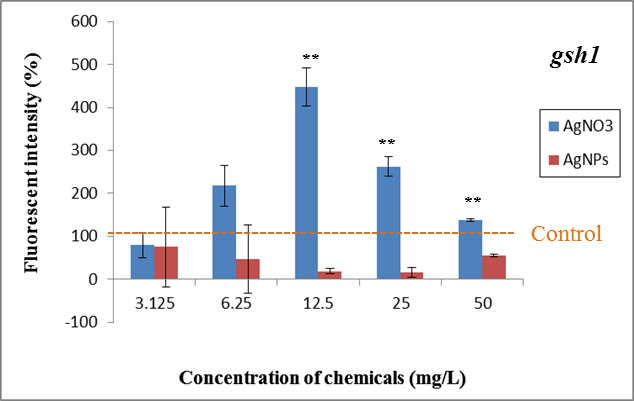
*
